# Internal carotid artery involvement and stroke risk in Takayasu arteritis: a case–control study

**DOI:** 10.1007/s00296-026-06075-7

**Published:** 2026-02-07

**Authors:** Hulya Odabasi Bukun, Ugur Uygunoglu, Esra Firat Senturk, Guzin Duru, Osman Kızılkılıç, Sinem Nihal Esatoglu, Melike Melikoglu, Emire Seyahi

**Affiliations:** 1 Department of Internal Medicine, Cerrahpasa Medical School, Istanbul University-Cerrahpasa, Istanbul, Turkey; 2 Department of Neurology, Cerrahpasa Medical School, Istanbul University-Cerrahpasa, Istanbul, Turkey; 3 Division of Rheumatology, Department of Internal Medicine, Cerrahpasa Medical School, Istanbul University-Cerrahpasa, Istanbul, Turkey; 4 Department of Pediatrics, Washington University, St Louis, USA; 5 Department of Neuroradiology Cerrahpasa Medical School, Istanbul University-Cerrahpasa, Istanbul, Turkey

**Keywords:** Takayasu arteritis, Stroke, Carotid stenosis, Cerebrovascular Disorders, Vasculitis

## Abstract

Stroke represents a major complication in Takayasu arteritis (TA). We aimed to determine clinical characteristics and neurological outcomes in TA patients with stroke compared to those without. We retrospectively analyzed 35 patients (27F/8 M) with documented stroke to 50 consecutive patients (47F/3 M) without stroke followed by the Istanbul University-Cerrahpasa Medical Faculty. Demographic data, clinical manifestations, arterial involvement patterns, treatments, and neurological outcomes were evaluated. Disability was assessed using the Expanded Disability Status Scale (EDSS), Barthel Index, and Modified Rankin Scale. Mean age at diagnosis among patients with stroke and non-stroke was similar (38.5 ± 10.7 vs. 35.6 ± 11.6 years). The mean age at stroke was 43.1 ± 10.3 years. Patients with stroke were more likely to be male (22.9% vs. 6.0%, p = 0.023). Strokes were predominantly ischemic (91.4%), affecting anterior circulation (82.8%) with left hemisphere predominance (72.4%). Internal carotid artery (ICA) involvement was significantly associated with stroke (right ICA: 51.4% vs 18.0%, p = 0.001; left ICA: 37.1% vs 18.0%, p = 0.047), while abdominal aorta involvement seemed to be protective (20.0% vs 42.0%, p = 0.028). Male gender (OR = 5.70, p = 0.038) and any ICA involvement (OR = 5.98, p = 0.004) were identified as independent predictors of stroke. Importantly, 40% experienced stroke as the initial TA manifestation. Among those developing stroke after TA diagnosis, 85.7% were already receiving immunosuppression and 47.6% antiplatelet therapy. Stroke patients demonstrated significant disability (mean EDSS: 3.63 ± 3.36 vs 0.02 ± 0.14, p < 0.001) and 11.4% mortality, median 5 years after stroke. Male patients and those with ICA involvement face the highest risk for stroke in TA. Long-term consequences are devastating with increased mortality, severe disability and high recurrence rates. The failure of immunosuppressive therapy to prevent stroke in the majority of treated patients, combined with substantial perioperative mortality, stress the inadequacy of current management strategies.

## Introduction

Takayasu arteritis (TA) is a chronic granulomatous large-vessel vasculitis that primarily affects the aorta and proximal part of its major branches [1–4]. The disease typically begins with panarteritis leading to chronic progressive wall thickening and variable degrees of stenosis or occlusion. The management of TA generally involves immunosuppressive therapy to control inflammation, with glucocorticoids being the cornerstone of initial treatment [3, 4]. Revascularization procedures, including bypass surgery and endovascular interventions, are considered for severe stenotic lesions causing persistent ischemic symptoms refractory to medical therapy [5, 6].

Neurological manifestations are common in TA patients particularly when aortic arch and carotid vessels are involved [7–13]. These include vertigo, headache, visual disturbances, transient ischemic attacks (TIA), and stroke. Stroke represents one of the most serious complications of TA, affecting approximately 10–20% of patients according to a meta-analysis [14]. The risk is estimated to be seven times higher than in the general population, placing TA among the leading causes of young stroke [13]. Despite its recognition as a major cause of morbidity and mortality in TA [15–20], several key aspects of stroke in this population remain poorly defined or remain limited to small case series. Using a well-characterized cohort at a tertiary referral center, we conducted a comparative analysis between patients with and without stroke to investigate the clinical characteristics, radiological features and treatment modalities.

## Methods

### Study design and population

We identified 35 patients with documented stroke (27F/8M) as the case group and 50 patients without stroke (47F/3M) who were consecutively registered at the Istanbul University-Cerrahpasa, Cerrahpasa Medical Faculty Rheumatology Outpatient Clinic between 2018 and 2021.

### Inclusion/exclusion criteria

Inclusion criteria: All patients were ≥ 18 years at diagnosis and met the 1990 American College of Rheumatology (ACR) classification criteria for TA [21]. All had documented cerebrovascular event (ischemic stroke or intracranial hemorrhage) confirmed by neuroimaging.

Exclusion criteria: Those with alternative etiology for stroke (cardioembolic source, atherosclerotic disease without vasculitis involvement and those with insufficient medical records or imaging for adjudication were not included in the study.

### Demographic and clinical data collection

Demographic and clinical data were collected from medical records, including age, gender, age at TA diagnosis, delay in diagnosis, presenting symptoms, comorbidities, and cardiovascular (CV) risk factors. The presence of comorbidities (diabetes, hypertension, ischemic heart disease, asthma, chronic obstructive pulmonary disease) was recorded. Other risk factors such as dyslipidemia, smoking status, and family history of CV diseases and stroke were also documented.

### Assessment of arterial involvement

Arterial involvement was evaluated using imaging studies including magnetic resonance imaging (MRI) and angiography (MRA), computed tomography angiography (CTA), and conventional angiography. The following arterial segments were assessed: common carotid arteries (CCA), internal carotid arteries (ICA), external carotid arteries (ECA), subclavian arteries (SBC), vertebral arteries, thoracic aorta, abdominal aorta, and renal arteries. Occlusion was defined as complete absence of luminal flow. ICA involvement was defined as wall thickening, stenosis, or occlusion affecting any segment of the internal carotid artery on CTA, MRA, or conventional angiography. Our imaging assessments were performed by expert neurologist and neuro-radiologist experienced in large-vessel vasculitis.

### Stroke definition and neurological evaluation

Stroke was defined as a sudden onset of focal neurological deficit lasting more than 24 h with evidence of cerebral infarction or hemorrhage on neuroimaging. For patients with stroke, detailed information was collected regarding the time of stroke relative to TA diagnosis, stroke type (ischemic or hemorrhagic), location (anterior or posterior circulation, right or left hemisphere), treatments received during the stroke period.

All cases and controls underwent neurological examination by a neurologist (U.U.). Neurological disability was assessed using the Expanded Disability Status Scale (EDSS), which ranges from 0 (normal neurological examination) to 10 (death due to neurological causes) [22]. Independence in daily activities was evaluated using the Barthel Index [23] and Modified Rankin Scale (mRS) [24]. For Barthel index, total possible scores range from 0 to 20, with lower scores indicating increased disability. Modified Rankin Scale is graded on the scale of 0–6: 0 means no symptoms at all, while 6 signifies death. Severe neurological disability was described as: EDSS ≥ 6.0 or mRS ≥ 4 or Barthel Index ≤ 12.

Since no validated neurological outcome scale currently exists specifically for TA or other large vessel vasculitides with CNS involvement, we used other measures. EDSS was indeed developed for multiple sclerosis, and mRS and Barthel Index are more commonly applied in stroke and general neurological disability assessment.

### Treatment assessment

Medical treatments were documented, including glucocorticoids, conventional disease-modifying antirheumatic drugs (DMARDs) (methotrexate, azathioprine, cyclophosphamide, leflunomide), biological DMARDs (infliximab, adalimumab, etanercept, certolizumab, golimumab, tocilizumab, rituximab), and anti-thrombotic therapy (antiplatelet agents, anticoagulants).

In patients who experienced stroke, the standard protocol included high-dose intravenous (IV) glucocorticoids during the acute phase, followed by conventional and or biologic DMARD therapy. Specifically, pulse methylprednisolone (1 g/day IV for 3–5 days) was administered, then transitioned to oral prednisolone (20–40 mg/day) with gradual tapering. Monthly IV cyclophosphamide (1 g every 4 weeks) was given for approximately six months. Maintenance therapy commonly involved biologic agents in combination with conventional DMARDs. Infliximab or tocilizumab was given priority at first place. Selection of consecutive biologics was individualized based on prior exposure and tolerability.

### Statistical analysis

Categorical variables were presented as frequencies and percentages, while continuous variables were presented as means and standard deviations. The Chi-square test or Fisher's exact test was used to compare categorical variables between groups. Continuous variables were compared between groups using the Mann–Whitney U test for non-normally distributed data. Formal sample size calculation was not performed given the retrospective design. The sample size was determined by the available cases at our institution. Multivariate logistic regression was done to identify variables associated independently with stroke. Covariates for the multivariate logistic regression model were selected based on clinical plausibility and univariate significance (p < 0.1). Variables included male gender, age at symptom onset, hypertension, hyperlipidemia, any ICA involvement and abdominal aorta involvement. Since the sample size (n = 85) and event number (n = 35) were small, Firth's penalized logistic regression was used. This method applies a penalty term to the likelihood function, producing less biased estimates and more reliable confidence intervals when events per variable are limited. A two-tailed p-values < 0.05 were considered statistically significant. All analyses were performed using SPSS Statistics version 26.0 (IBM Corp., Armonk, NY, USA).

### Ethical statement

The study protocol was approved by Ethics Committee of Istanbul University-Cerrahpasa, Cerrahpasa Medical Faculty (08/05/2020–60363). Written informed consent for publication of clinical images was obtained from relevant patients.

## Results

### Demographic and clinical characteristics

Demographic and clinical characteristics of the cases and controls were compared in Table 1. Patients with stroke were significantly more likely to be male (22.9% vs. 6.0%, p = 0.023). Patients with stroke were more likely to be older at symptom onset (36.1 ± 10.9 vs. 31.4 ± 10.8, p = 0.043) as well as at diagnosis (38.5 ± 10.7 vs. 35.6 ± 11.6, p = 0.162) compared to those without stroke. The mean delay in diagnosis (2.5 ± 3.8 vs. 4.2 ± 6.9 years, p = 0.245) was similar between the groups. The mean age at stroke was 43.1 ± 10.3 years and median time from symptom onset to stroke was 3.5 years [IQR: 0–11].

**Table 1:** Demographic and Clinical Characteristics of Patients with and without Stroke

Characteristic	Stroke (*n* = 35)	No stroke (*n* = 50)	*p* value
Demographics			
Male, *n* (%)	8 (22.9%)	3 (6.0%)	0.023*
Age at TA diagnosis, mean ± SD, years	38.5 ± 10.7	35.6 ± 11.6	0.162
Age at symptom onset, mean ± SD, years	36.1 ± 10.9	31.4 ± 10.8	0.043*
Delay in diagnosis, mean ± SD, years	2.5 ± 3.8	4.2 ± 6.9	0.244
Follow-up duration, mean ± SD, years	14.7 ± 9.1	13.7 ± 6.6	0.982
Age at stroke, mean ± SD, years	43.1 ± 10.3	-	-
CV risk factors and comorbidities, *n* (%)			
Hypertension	27 (77.1)	32 (64.0)	0.196
Diabetes Mellitus	2 (5.7)	3 (6.0)	0.956
Hyperlipidemia	22 (62.9%)	19 (38.0%)	0.042*
Smoking	9 (25.7)	12 (24.0)	0.857
Cardiovascular disease	4 (11.4)	5 (10.0)	0.833
Comorbid diseases	5 (14.3)	3 (6.0)	0.197
Familial history of CV disease	11 (31.4)	20 (40.0)	0.419
Family history of stroke	10 (28.6)	8 (16.0)	0.163
Concomitant inflammatory disease†	9 (25.7)	11 (22.0)	0.751
Symptoms, *n* (%)			
Limb Claudication/Pain/Numbness	27 (77.1)	37 (74.0)	0.741
Carotidynia	6 (17.1)	7 (14.0)	0.692
Weakness/Fatigue	15 (42.9)	23 (46.0)	0.774
Headache	15 (42.9)	26 (52.0)	0.406
Transient ischemic attacks	17 (48.6)	14 (28.0)	0.052
Vertigo	17 (48.6)	17 (34.0)	0.177
Speech disturbance	11 (31.4)	2 (4.0)	0.001*
Facial paralysis	8 (22.9)	0	< 0.001*
Transient monocular blindness	8 (22.9)	4 (8.0)	0.053
Hearing loss	7 (20.0)	5 (10.0)	0.193
Disability scores, mean ± SD			
EDSS	3.63 ± 3.36	0.02 ± 0.14	< 0.001
Barthel index	16.6 ± 4.6	20.0 ± 0.0	0.001
Modified rankin scale	1.83 ± 1.60	0.06 ± 0.42	< 0.001

A patient may have more than one concomitant condition

*SD* standard deviation, *TIA* transient ischemic attack, *CV* cardiovascular, *EDSS* expanded disability status scale

*Statistically significant (*p* < 0.05)

† ankylosing spondylitis or psoriasis (*n* = 10), inflammatory bowel disease (*n* = 7), amyloidosis (*n* = 1), glomerulonephritis (n = 1) and pericarditis (*n* = 1), hidradenitis suppurativa (*n* = 1), rheumatoid arthritis (*n* = 1) and interstitial lung disease (*n* = 1)

Both groups were comparable regarding CV risk factors, including hypertension (77.1% vs. 64.0%), diabetes mellitus (5.7% vs. 6.0%), prior history of CV diseases (11.4% vs. 10.0%), comorbid diseases (17.1% vs. 12.0%), and smoking (25.7% vs. 24.0%) (Table 1). Family history of premature CV disease and stroke were also similar between the groups. On the other hand, it was noted that, hyperlipidemia (62.9% vs 38.0%, p = 0.042) was more common in the stroke group. Considerable number of patients from both groups had one or more concomitant inflammatory diseases (25.7% vs. 22.0%). These were most commonly ankylosing spondylitis (AS) (n = 9) and inflammatory bowel disease (IBD) (n = 7) and less frequently amyloidosis, pericarditis, psoriasis, hidradenitis suppurativa, rheumatoid arthritis, interstitial lung disease, and glomerulonephritis, each being present in one patient.

Upper or lower extremity claudication was the most common symptom in both groups (77.1% vs. 74.0%). This was followed by weakness/fatigue, vertigo, hearing loss, headache, and carotidynia which were balanced across the study groups. TIA (48.6% vs. 28.0%, p = 0.052), transient mono-ocular blindness (TMB) episodes (22.9% vs. 8.0%, p = 0.053) and speech disturbance (31.4% vs. 4.0%, p = 0.001) were more common in patients with stroke compared to those without stroke, although only the latter differed significantly. Facial paralysis was only found in stroke patients (22.9%).

### Patients with stroke before vs. after TA diagnosis

Of the stroke patients, 14 (40.0%) initially presented with stroke prior to their TA diagnosis and were subsequently followed in our department whereas the remaining 21 patients (60%) developed stroke at a mean of 9.5 ± 7.9 years after the initial diagnosis of TA while under follow-up at our rheumatology outpatient clinic.

This distribution enabled analysis of stroke both as an initial manifestation and as a subsequent complication of TA. We compared these two subgroups whether to see if there are any differences regarding demographic or clinical characteristics. Patients who experienced stroke before diagnosis were younger [39.4 ± 10.2 vs. 45.2 ± 11.8, p = 0.138) and more frequently presented with upper or lower extremity claudication (100% vs. 61.9%, p = 0.014). Other clinical features, comorbidities, arterial involvement, follow-up neurological findings, disability scores, and mortality rates were similar between groups (data not shown).

### Arterial involvement

**Fig. 1:**
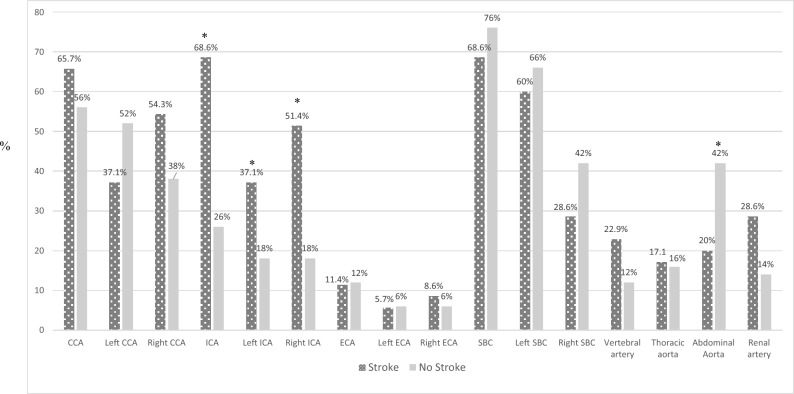
Patterns of arterial involvement in Takayasu Arteritis patients with and without stroke. Showing the percentage of affected vessels in patients with and without stroke. Note the significantly higher involvement of ICA (both left and right ICA) in stroke patients (p < 0.05) and higher abdominal aorta involvement in non-stroke patients (p < 0.05). *CCA* common carotid artery, *ICA* any internal carotid artery, *ECA* external carotid artery, *SBC* subclavian artery

**Fig. 2:**
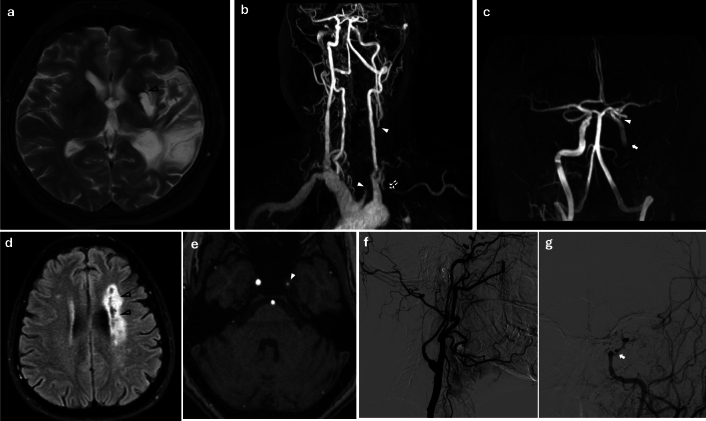
Cranial MRI and MRA scans of Takayasu arteritis patients who presented with stroke. Upper panel shows supra-aortic involvement, while lower panel shows supra-aortic along with medium-sized intracranial arterial vasculitis. (upper panel): A 48-year-old female with a 10-year history of Takayasu arteritis presented with progressive right hemiplegia. a. Axial T2-weighted MRI demonstrates a large hemispheric chronic deep white matter (WM) infarction involving the left capsule-striatal region (black-lined arrowhead) as well as the posterior temporal lobe and angular gyrus. b. Contrast-enhanced MR angiography displayed the chronic occlusion of the left common carotid artery (CCA), extending approximately 1 cm from its origin at the aortic arch to the bifurcation level (white arrowheads). The non-enhancing segment between the two arrowheads indicates the extent of the long-segment CCA occlusion. The chronic occlusion of the left subclavian artery proximal segment is also visualized (dashed arrow). c. Time-of-Flight (TOF) MR angiography without contrast shows a normal-appearing intracranial segment of the left internal carotid artery (ICA) (arrowhead). However, no flow signal is detected in the distal cervical and petrous segments of the left ICA (downstream of the white arrow), consistent with retrograde flow originating from the contralateral, normal side through the anterior communicating artery. (lower panel): The 57-year-old female patient, previously known as Takayasu arteritis, presented with right hemiparesis with paraphasia. d. axial FLAIR image shows a wide zone of leukomalacia with central rarefaction involving the supraventricular WM and corona radiata, consistent with large deep WM infarcts reflecting the water-shed zones (black-lined arrowheads). e. TOF MR angiography depicted marked concentric intima-media thickening with hemodynamically significant narrowing in the cavernous segment of the left ICA (white arrowhead), representing another focus of vasculitic involvement. f. Subsequent Digital Subtraction Angiography (DSA) from the left CCA injection displayed severe narrowing and contour abnormalities along the entire cavernous-supra-clinoid segment of ICA (black arrows), suggesting vasculitic involvement. Enlarged branches of the left external carotid artery, such as the internal maxillary artery and the prominent left middle meningeal artery, reconstitute the salvage collateralizations beyond the stenotic left ICA segment. g. Further DSA with selective catheterization of the left ICA showed steno-occlusive narrowing of the left cavernous ICA after removing the collateral contributions effect (white arrow)

Analysis of arterial involvement revealed significant differences between the two groups (Fig. 1). ICA involvement (68.6% vs. 26.0%, p < 0.001) was significantly more common in patients with stroke (Fig. 1). This was true for both right (51.4% vs. 18.0%, p = 0.001) and left ICA involvement (37.1% vs. 18.0%, p = 0.047). Conversely, abdominal aorta involvement was significantly more frequent in patients without stroke (20.0% vs. 42.0%, p = 0.028). Besides that, no significant differences were observed in the involvement of CCA, ECA, SBC, vertebral arteries, thoracic aorta, or renal arteries. Intracranial artery involvement was detected by conventional angiography in only one of six patients who underwent this procedure (Fig. 2 lower panel).

### Stroke characteristics

Of the 35 patients with stroke, 32 (91.4%) had ischemic stroke and 3 (8.6%) had hemorrhagic stroke, including one case of subarachnoid hemorrhage.

MRI and MRA images were both available in 34 patients who experienced stroke. Evaluation of hemispheric infarct localization revealed that anterior circulation involvement was more prevalent (n = 29, 82.8%) compared to posterior circulation involvement (n = 5, 14.8%). Among those with anterior circulation infarcts, the left middle cerebral artery (MCA) territory was more frequently affected than the right (left: n = 21, 72.4%; right: n = 8, 27.6%) (Fig. 2). To note, laterality was not associated significantly with left or right ICA involvement (left MCA territory-left ICA 10/21 vs right MCA territory-right ICA: 5/8, p = 0.473).

Among three patients who experienced stroke there were isolated renal artery or abdominal aorta involvement, without any carotid or supra-aortic involvement. No patent foramen ovale or any other cardiac causes were identified either. Neuroimaging studies were also reviewed for other potential neurological comorbidities. None of the patients had concomitant neuro-degenerative disorders. No findings suggestive of multiple sclerosis -like lesions, Behçet-like lesions, or cerebral venous sinus thrombosis were identified. Cranial MR imaging was available in 45 patients in the non-stroke and no apparent significant lesion was found.

### Independent variables associated with stroke

Multivariate logistic regression analysis with Firth correction identified independent predictors of stroke (Table 2). After adjusting for age at symptom onset, hypertension, hyperlipidemia, and abdominal aorta involvement, two variables remained independently associated with stroke: male gender (OR = 5.70, 95% CI 1.10–29.41, p = 0.038) and any internal carotid artery involvement (OR = 5.98, 95% CI 1.77–20.26, p = 0.004). It has to be noted, the wide confidence intervals reflect the relatively small sample size.

**Table 2:** Multivariate logistic regression† analyzing variables associated with stroke

Variable	Odds ratio	95% confidence intervals	*p* value
Male gender	5.70	1.10–29.41	0.038*
Age at symptom onset	0.99	0.94–1.04	0.617
Hypertension	2.68	0.68–10.59	0.160
Hyperlipidemia	2.41	0.75–7.72	0.138
Any internal carotid artery involvement	5.98	1.77–20.26	0.004*
Abdominal aorta involvement	0.63	0.19–2.13	0.456

*Statistically significant (*p* < 0.05)

^†^The Firth correction was done

### Treatment

(i) Medical treatment
  Treatment records during the stroke period revealed that 18 (85.7%) of the 21 patients who developed stroke after TA diagnosis were receiving immunosuppressive therapy at the time of stroke. The treatments included prednisolone (n = 15, 83.3%), methotrexate (n = 5, 27.8%), infliximab (n = 5, 27.8%), azathioprine (n = 8, 44.4%), and cyclophosphamide (n = 2, 11.1%). Ten patients (47.6%) developed stroke while on antiplatelet therapy.
  After the stroke event, treatment was intensified in most patients as shown in Table 3. The use of biological DMARDs was similar in frequency between the stroke (68.6%) and non-stroke (58.0%) groups (p = 0.369). Among the biologics, anti-TNF agents were the most commonly used in both groups (65.7% vs. 54.0%, p = 0.371). All patients had used either glucocorticoids (97.1% in stroke vs. 96.0% in non-stroke patients, p = 1.000) and or conventional DMARDs (97.1% vs. 94.0%, p = 0.640), with methotrexate and azathioprine being frequently prescribed.

**Table 3:** Treatment regimens in Takayasu arteritis patients

	Stroke (*n* = 35)	No stroke (*n* = 50)	*p* value
Medical treatment			
Biological DMARDs, *n* (%)	24 (68.6)	29 (58.0)	0.369
Anti-TNF agents	23 (65.7)	27 (54.0)	0.371
Tocilizumab	4 (11.4)	7 (14.0)	0.728
Rituximab	5 (14.3)	5 (10.0)	0.546
Conventional DMARDs, *n* (%)	34 (97.1)	47 (94.0)	0.640
Methotrexate	16 (45.7)	32 (64.0)	0.094
Azathioprine	17 (48.6)	29 (58.0)	0.391
Cyclophosphamide	13 (37.1)	14 (28.0)	0.373
Leflunomide	3 (8.6)	2 (4.0)	0.378
Glucocorticoids, *n* (%)	34 (97.1)	48 (96.0)	1.000
Antithrombotic therapy, *n* (%)			
Aspirin	25 (71.4)	21 (42.0)	0.007*
Anti-hyperlipidemic medication use	22 (62.9)	19 (38.0)	0.024*
Anticoagulant	7 (20.0)	1 (2.0)	0.005*
Surgery and or vascular intervention, *n* (%)			
Any type of surgery and/or vascular intervention	18 (51.4)	13 (26.0)	0.016
Renal artery stenting and or balloon angioplasty	4 (11.4)	5 (10.0)	
Aorto-bifemoral/biiliac bypass or iliac artery stenting	0	8 (16)	
SBC or aorto-carotid bypass or carotid/SBC artery stenting	13 (37.1)	2 (4)	
Coronary artery stenting /bypass grafting	3 (8.6)	1 (2)	
Bentall procedure or aortic graft replacement	2 (5.7)	2 (4)	

*DMARDs* disease-modifying antirheumatic drugs, *TNF* tumor necrosis factor, *SBC* subclavian

*Not using any immunosuppressive agents or glucocorticoids

*Statistically significant (*p* < 0.05)
  Antiplatelet therapy was prescribed significantly more often in the stroke group compared to the non-stroke group (71.4% vs. 42.0%, p = 0.007). Anticoagulant therapy (20.0% vs. 2.0%, p = 0.005) and lipid-lowering medications (62.9% vs. 38.0%, p = 0.024) were also more frequently utilized in the stroke group.
(ii) Surgery or vascular intervention
  As depicted in Table 3, surgical or vascular interventions were significantly more frequent among TA patients who experienced stroke compared to those without stroke (51.4% vs. 26.0%, p = 0.016). Carotid-subclavian bypass, aorto-carotid bypass, or carotid/subclavian artery stenting was performed in 37.1% of the stroke group versus only 4% of the non-stroke group. Conversely, aorto-bifemoral or aorto-biiliac bypass and iliac artery stenting were only observed in the non-stroke group (16.0%). Other procedures, including renal, coronary, and aortic interventions, showed no significant differences between groups.
  It has to be noted that, six patients (17.1%) developed stroke in the post-operative period. Five of these events occurred following carotid surgery, and one occurred after a femoral artery intervention. Of these, three died after carotid surgery.

### Stroke recurrences, epileptic seizures, neurological disability and death

At the time of study entry, four patients (3F/1M) (11.4%) were known to be deceased. The remaining 31 patients were alive at a mean of 13.2 ± 8.9 years after their stroke. Of the four patients who died overall, three deaths occurred in the postoperative period following carotid surgery.

Neurological evaluation was performed at a median of 5 years (IQR: 3–11) following stroke in the stroke group and at a median of 7.0 years (IQR: 4.0–11) following diagnosis in the control group. There was significant disability among stroke patients. Patients with stroke had significantly higher scores on disability measures, including EDSS (3.63 ± 3.36 vs. 0.02 ± 0.14, p < 0.001), modified Rankin Scale (1.83 ± 1.60 vs. 0.06 ± 0.42, p < 0.001), and lower scores on the Barthel index (16.6 ± 4.6 vs. 20.0 ± 0.0, p = 0.001), indicating greater disability (Table 1). Apart from four who had died (11.4%), a total of 9 patients (6F/3M) (25.7%) had severe disability.

Six patients (17.1%) experienced recurrences (a least two consecutive stroke episodes), while others had a single stroke (recurrent stroke incidence rate per 100 person-years was calculated as 1.17 (95% CI: 0.43–2.54). Six patients (17.1%) experienced epileptic seizures; four of them were receiving antiepileptic treatment. Post-stroke epilepsy incidence rate per 100 person-years was found 1.17 (95% CI: 0.43–2.54).

## Discussion

This cross-sectional retrospective study revealed that among patients with stroke there is a predilection for male patients (OR: 5.70) and a strong association with ICA involvement (OR: 5.98). Primarily ischemic infarcts were observed, localized in the MCA territory and frequently affecting the left hemisphere. Stroke was the initial clinical manifestation in 40% of cases, while in the remaining patients, it occurred despite ongoing immunosuppressive and antiplatelet treatments. Surgical interventions involving arcus aorta resulted in severe consequences. Finally, stroke left devastating marks: a median of 5 years (IQR: 3–11) after stroke, 4 patients (11.4%) had died and 9 (25.7%) were severely disabled. Our findings suggest that current strategies for both preventing and managing stroke in TA patients remain insufficient.

We only investigated patients with a definitive diagnosis of stroke, intentionally excluding TIA due to the diagnostic uncertainty [25–27]. Whereas, several researchers consider TIA similar to stroke as part of the cerebrovascular diseases [7–11, 14, 20]. In this regard, TIAs, especially transient monocular blindness is strongly associated with subsequent stroke. Thus, while our approach ensured diagnostic precision, we may have underestimated the true burden of cerebrovascular involvement in TA.

Our study suggests that male gender may be a risk factor for stroke in TA. This observation contrasts with the overall female predominance in TA but aligns with the higher stroke risk in males in the general population [28]. Male overrepresentation among stroke cases in TA has been noted by others, as well [12, 20]. It is possible that regional or genetic differences modulate this risk, or that male patients in clinic cohorts have more aggressive disease requiring referral. We have already reported that TA is associated with premature atherosclerosis independent of traditional CV risk factors [29–31]. Therefore, another consideration is that male TA patients – who often have higher rates of smoking and metabolic risk – could have an added atherosclerotic propensity contributing to stroke on top of vasculitic injury that is already prone to accelerated atherosclerosis. This gender dimorphism in stroke characteristics warrants further investigation.

The strong association between ICA involvement and stroke in our cohort supports previous research indicating that involvement of the aortic arch and its branches, particularly the carotid arteries, is a major risk factor for cerebrovascular events in TA [8–12]. Conversely, abdominal aorta involvement was significantly more common in patients without stroke, suggesting a potentially distinct disease phenotype with lower cerebrovascular risk. Consistent with our findings, prior studies have reported higher frequencies of ICA lesions in TA patients with stroke compared to those without [7, 10, 12]. Interestingly, the right ICA tend to be affected more often than the left [32, 33]. In line with that, very recently, machine learning models have identified ICA pathology as a key predictor for severe ischemic complications [34]. It is also worth noting that intracranial artery involvement, though less common, can also occur in TA and contribute to stroke risk [32, 33, 35, 36]. And very rarely, as seen in a single patient in our cohort, tandem extracranial and intracranial disease may develop in severe cases (Fig. 2 lower panel). Clinically, these findings stress the need for comprehensive vascular imaging in TA patients to fully assess stroke risk.

Moreover, consistent with previous studies, we observed that the majority of strokes in our TA patients involved anterior circulation more specifically the MCA territory (82.9%). The left-sided predominance of infarcts represents a novel finding that may relate to anatomical factors and also to immune regulation [37–39]. The direct branching of the left CCA from the aortic arch may predispose to greater hemodynamic stress and atherosclerotic burden on the left side. This lateralization has been observed in atherosclerotic stroke populations [39], but has not been previously reported in TA.

The predominance of left MCA territory infarction despite more frequent right ICA involvement is also intriguing. This could be due to several factors: (1) differential collateral capacity between hemispheres; (2) variation in the extent or hemodynamic significance of stenosis not captured by binary involvement classification; (3) embolic mechanisms from aortic arch or left common carotid involvement; or (4) chance finding given small numbers. This observation warrants investigation in larger cohorts with detailed hemodynamic assessment.

Hemorrhagic stroke is rare in TA, often related to long-standing hypertension or aneurysm rupture. Ischemic events accounted for the vast majority of strokes in our cohort, consistent with prior reports [20, 35]. Beyond simple hemodynamic compromise from stenosis, thromboembolism emerges as another possible mechanism contributing to stroke in TA. Imaging studies describe both watershed infarctions from progressive stenosis and large lobar infarctions from thromboembolism originating in inflamed vessel segments [35, 40–44]. While the precise mechanisms underlying in situ thrombosis remain unclear, it is possible that it is promoted through active arterial wall inflammation, endothelial dysfunction and accelerated atherosclerosis [29–31, 45, 46].

Importantly, 40% of our patients who suffered stroke had cerebrovascular ischemia as the initial manifestation of TA. A similar observation was noted by several others, though with changing proportions ranging from 15 to 66.7% [9–12, 19, 20]. This aligns also with a large national population survey indicating that 56.2% develop stroke within 6 months of diagnosis [13]. There was a high proportion of patients presenting with claudication symptoms in our cohort that probably prompted clinicians to consider TA. However, this cannot be taken for granted, as classic peripheral signs may be absent in these patients as shown by Misra et al. [20], adding to the diagnostic challenge. Altogether these findings suggest that vascular involvement in TA can progress silently until luminal compromise or inflammation reaches a critical threshold, precipitating stroke. Another important point is that these temporal patterns may suggest two distinct stroke phenotypes in TA: early strokes related to active inflammation and late strokes reflecting established vascular damage. The fact that 85.7% who developed stroke after TA diagnosis were already using immunosuppressive agents supports this assumption.

Our findings regarding treatment patterns reflect current management approaches for TA, with a high proportion of patients receiving biological DMARDs [3, 4]. The significantly higher use of antiplatelet, anticoagulant and anti-hyperlipidemic therapy in the stroke group is expected as part of secondary prevention strategies [47]. However, the occurrence of stroke, high relapse rate and increased mortality despite these therapies suggest that current regimens may incompletely mitigate cerebrovascular risk in some patients. This observation could be limited by indication bias and may not imply treatment inefficacy. Another explanation could be the concept of "smoldering inflammation"—persistent vascular inflammation despite apparent clinical remission – which may explain worsening despite treatment [48, 49]. Angiographic progression despite clinical remission in TA is rather well known [50]. This discrepancy between systemic inflammatory markers and local vascular inflammation represents an important phenomenon in the pathogenesis of TA [49–51]. Vascular damage may be irreversible in some cases, and prothrombotic or hemodynamic factors can persist even in inactive disease. Additionally, advanced atherosclerosis, diffuse arterial calcification and adventitial fibrosis could be other potentially aggravating factors [29–31, 52].

We observed that surgery was associated with severe outcome: six patients developed stroke predominantly after carotid intervention and three died due to the surgery related complications. This underscores the high risks of revascularization in TA [53]. Recent meta-analyses comparing surgical versus endovascular interventions reveal complex trade-offs: while open surgery demonstrates superior long-term patency, endovascular procedures carry lower immediate stroke risk [54, 55]. However, the critical determinant remains disease activity at intervention and contemporary guidelines emphasize achieving disease remission before elective revascularization, with serial imaging to confirm arrested progression [3, 4]. The complexity of vascular procedures, and center-specific experience are also potential contributing factors. While these findings represent associations and do not establish causality, caution is required before planning vascular intervention in TA.

The co-occurrence of inflammatory diseases (25.7%), particularly ankylosing spondylitis and inflammatory bowel disease, confirms our previously reported associations [56] and may reflect shared genetic susceptibility between TA and these diseases.

Stroke recurrence (17.1%) and post-stroke epilepsy (17.1%) in our cohort, despite combined immunosuppressive and antiplatelet therapy, highlight a major gap in secondary prevention. These rates exceed those seen in the general population but lower than that found in prior TA cohorts, where French multicenter surveys reported recurrence rates of 35–54% and epilepsy incidence of 24% [10, 19]. Our study also sheds light on the neurological sequelae of stroke in TA patients. Speech disturbance, facial paralysis, and visual disturbances were significantly more common in patients with stroke. The mean EDSS score of 3.63 with 25.7% experiencing severe disability indicate that these patients have significantly impaired quality of life. The 11.4% mortality rate, predominantly from surgical complications, emphasizes stroke's serious prognosis in TA.

Finally, our study demonstrated that these patients carried significant long-term disability and mortality. This finding suggests a critical need for quality rehabilitation services. However, recent evidence reveals significant barriers [57, 58]. These limitations are particularly devastating for young TA stroke survivors who face decades of disability during their productive years. The combination of young-onset stroke, significant disability burden, and inadequate access to evidence-based rehabilitation resources amplifies the long-term impact of cerebrovascular complications in TA.

Future research should include multicenter, prospective registries to overcome sample size limitations inherent to single-center studies of this rare disease. Standardized protocols for neuroimaging and disease activity assessment at stroke onset would facilitate pooled analyses. Optimal timing of revascularization, and the role of advanced imaging (vessel wall MRI) in identifying high-risk lesions may represent important directions.

This study has several limitations. The retrospective observational design introduces potential selection and recall bias and precludes causal inference. All reported associations should be interpreted with caution. The limited sample size restricts statistical power, particularly for subgroup analyses . Given multiple comparisons, these findings carry elevated type I error risk and should be interpreted as hypothesis-generating. The single-center nature may limit generalizability, though it ensured consistent management protocols. Imaging modalities and treatment approaches evolved over the study period, potentially affecting comparisons. Inflammatory markers and disease activity scores were not reported due to the incomplete data. Neurological outcome scales that are used in the study (EDSS, mRS and Barthel Index) have not been validated in vasculitis populations. The outcome "stroke despite immunosuppression" is susceptible to selection and timing biases. Importantly, comparison of mortality between patients with and without stroke is subject to immortal time bias, as patients who experienced stroke years after diagnosis had, by definition, survived until that event. This may attenuate observed mortality differences between groups. Accordingly, we present mortality as a descriptive outcome rather than a formal comparative analysis, and suggest caution against causal interpretation. Nevertheless, the study also has strengths such as comprehensive neurological assessments by a dedicated neurologist and detailed stroke characterization with proper neuroimaging.

## Conclusions

Stroke in TA shows distinctive characteristics: younger age at onset, predominant anterior circulation and left hemisphere involvement, and strong association with ICA disease. Male sex appears to have increased stroke risk despite the overall female predominance of TA. In a substantial subset of patients, stroke occurred as the initial manifestation of TA challenging timely diagnosis. The failure of current therapeutic approaches—evidenced by high rates of stroke despite treatment, peri-operative stroke occurence, increased mortality, recurrence, and post-stroke epilepsy—demands reconsideration of management strategies.

## Data Availability

The data that support the findings of this study are included in the article or uploaded as supplementary information. Additional data are available from the corresponding author upon request.
